# Supplementary material for: Nitric oxide is a host cue for Salmonella Typhimurium systemic infection in mice
Source: Commun Biol. 2023 May 9;6:501. doi: 10.1038/s42003-023-04876-1 (PMC10169850; doi:10.1038/s42003-023-04876-1)
Supplement: Supplementary file 2 — Supplementary Information [file 42003_2023_4876_MOESM2_ESM.pdf]

SUPPLEMENTARY INFORMATION

Nitric oxide is a host cue for *Salmonella* Typhimurium systemic infection in mice

Lingyan Jiang[1,2], Wanwu Li[1,2], Xi Hou[1,2], Shuai Ma[1,2], Xinyue Wang[1,2], Xiaolin Yan[1,2], Bin Yang[1,2], Di Huang[1,2], Bin Liu[1,2], Lu Feng [1,2,*]

[1] The Key Laboratory of Molecular Microbiology and Technology, Ministry of Education, Nankai University, Tianjin, China

[2] TEDA Institute of Biological Sciences and Biotechnology, Tianjin Key Laboratory of Microbial Functional Genomics, Nankai University, Tianjin, China

[*] Correspondence: Lu Feng (e-mail: fenglu63@nankai.edu.cn)

–**Supplementary Figures:**

Supplementary Fig. 1; Supplementary Fig. 2; Supplementary Fig. 3; Supplementary Fig. 4; Supplementary Fig. 5; Supplementary Fig. 6

–**Supplementary Tables:**

Supplementary Table 1; Supplementary Table 2; Supplementary Table 3; Supplementary Table 4; Supplementary Table 5

–**Supplementary Figures**

Supplementary Fig. 1

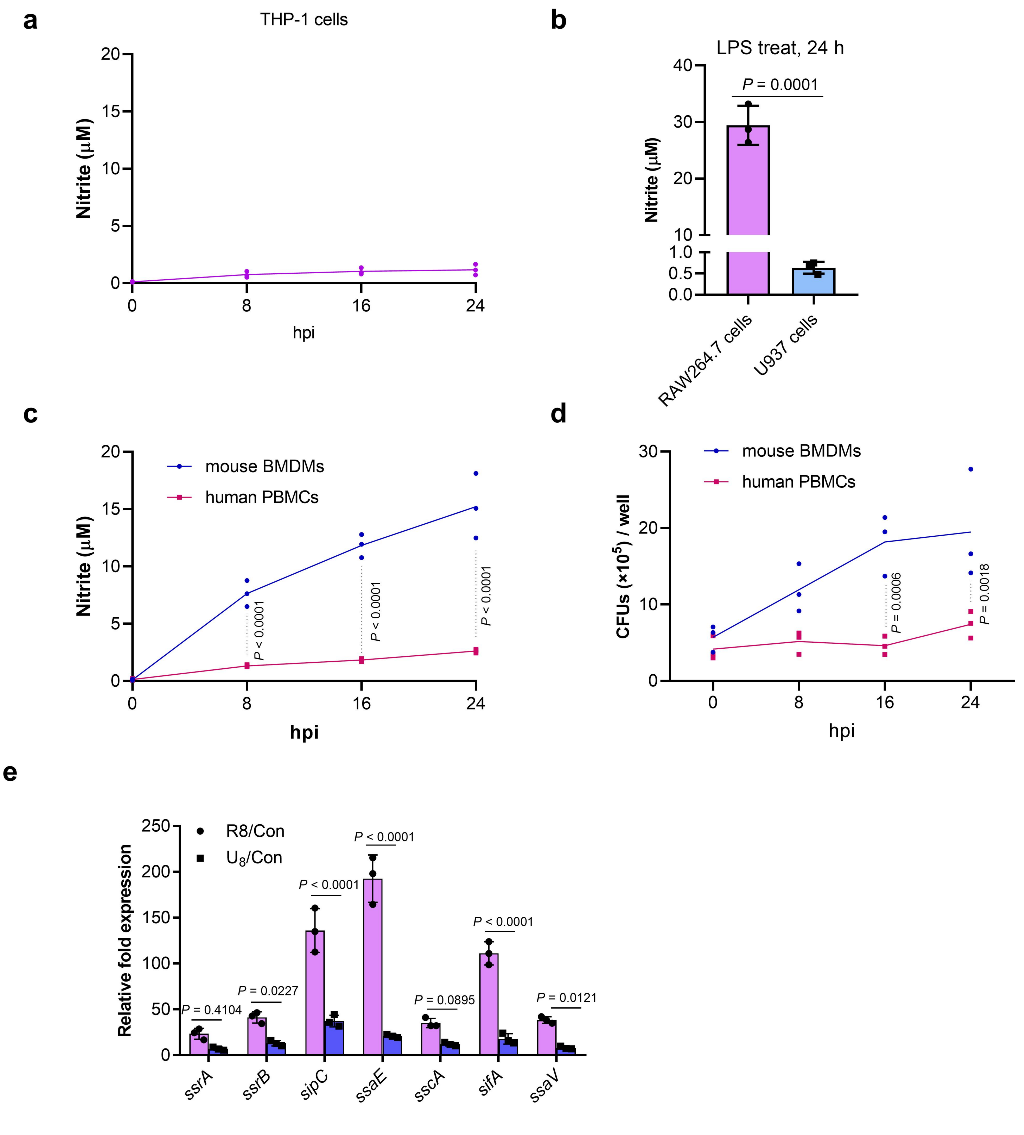

Supplementary Fig. 1 (related to Fig. 1) Host NO levels correlate positively with *S.* Typhimurium replication and SPI-2 expression levels in mouse macrophages. a, Nitrite production by human THP-1 cell lines left uninfected (0 hpi) or infected with *S.* Typhimurium wild-type strain ATCC 14028s (n = 3 independent experiments). Cells were infected at a MOI of 10, and nitrite levels in the supernatant were measured using Griess assays at the indicated time points. **b,** Nitrite production by

RAW264.7 cells and U937 cells stimulated with LPS (10 ng/mL) for 24 h (n = 3 independent experiments). **c**, Nitrite production by mouse primary bone-marrow-derived macrophages (BMDMs) and human peripheral blood mononuclear cells (PBMCs) left uninfected (0 hpi) or infected with *S.* Typhimurium wild-type (n = 3 independent experiments). **d,** Bacteria burden of *S.* Typhimurium wild-type in BMDMs and PBMCs (n = 3 independent experiments). Bacterial CFU ($\times 10^5$) /well (y axis) and time after addition of gentamicin (x axis) are indicated. **e,** Confirmation of RNA-seq results by qRT-PCR for seven SPI-2 genes (n = 3 independent experiments). RAW264.7 cells or U937 cells were infected with *S.* Typhimurium wild-type for 8 h, cells were then lysed and the intracellular bacteria were collected for RNA extraction and qRT-PCR (R_8: RAW264.7, 8 hpi; U_8: U937, 8 hpi). RNA extracted from bacteria in the RPMI-1640 medium was used as a control (Con). All data are presented as mean ± SD. *P* values were determined using two-tailed unpaired Student's *t*-test (**b**) or two-way ANOVA (**c**–**e**). hpi, hours post-infection. Source data are included in Supplementary Data 1.

Supplementary Fig. 2

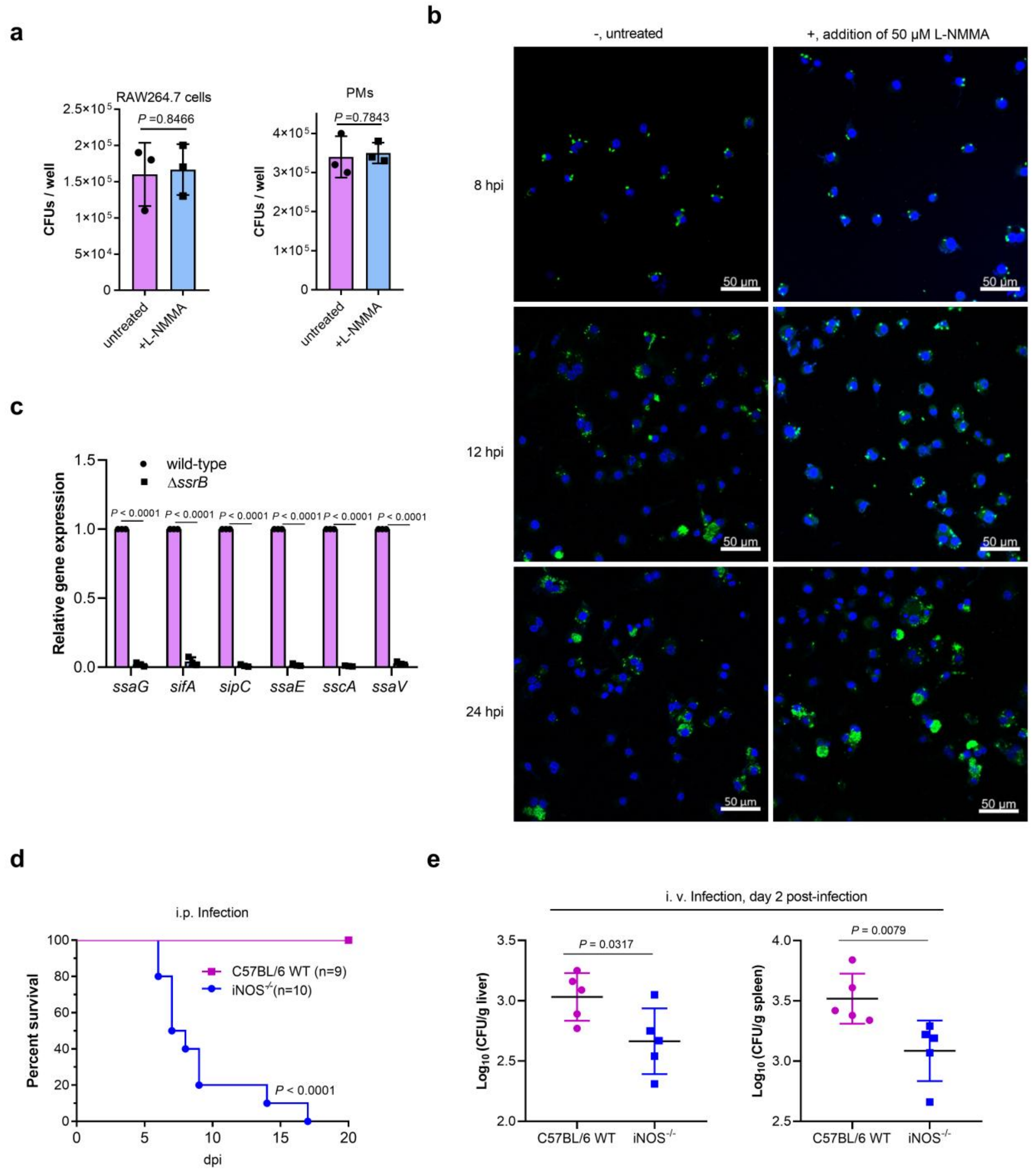

Supplementary Fig. 2 (related to Fig. 2) Lack of host NO reduced *S.* Typhimurium SPI-2 gene expression and bacterial replication in mouse macrophages, and reduced bacterial burden in mouse systemic organs at early infection stages. a, Uptake of *S.* Typhimurium by RAW264.7 cells and PMs derived from C57BL/6 mice in the presence or absence of 50 μM L-NMMA (n = 3 independent experiments). RAW264.7 cells and PMs were infected with wild-type *S.* Typhimurium at an MOI of 10. L-NMMA was added 2 h prior to infection. At 15 min

post-infection, the infected cells were washed three times with PBS, and intracellular bacteria were released for CFU enumeration. **b,** Representative immunofluorescence images of RAW264.7 cells infected with wild-type *S.* Typhimurium at 8, 12, and 24 hpi. RAW264.7 cells were seeded on coverslips and infected with wild-type *S.* Typhimurium at an MOI of 10. Infected cells were fixed at indicated time points and stained with anti-*S.* Typhimurium LPS antibody (green) and DAPI (blue) and then analyzed by confocal microscopy. Scale bars, 50 μm. **c**, qRT-PCR analysis of *ssaG*, *sifA*, *sipC*, *ssaE*, *sscA*, and *ssaV* mRNA levels in *S.* Typhimurium wild-type and *ssrB* mutant (n = 3 independent experiments). RNA was extracted from bacteria grown in N-minimal medium. **d,** Survival plots of C57BL/6 wild-type (WT) and iNOS$^{-/-}$ mice after intraperitoneal (i.p.) inoculation with ~5000 CFUs *S.* Typhimurium wild-type. n = 9 mice for WT, n =10 mice for iNOS$^{-/-}$. **e**, Bacterial counts recovered from the liver and spleen of C57BL/6 WT and iNOS$^{-/-}$ mice intravenously (i.v.) infected with ~500 CFUs *S.* Typhimurium wild-type at day 2 post-infection, n = 5 mice per group. All data are presented as mean ± SD. *P* values were determined using two-tailed unpaired Student's *t*-test (**a**), two-way ANOVA (**c**), log-rank Mantel-Cox test (**d**), or Mann-Whitney U test **(e)**. Source data are included in Supplementary Data 1.

Supplementary Fig. 3

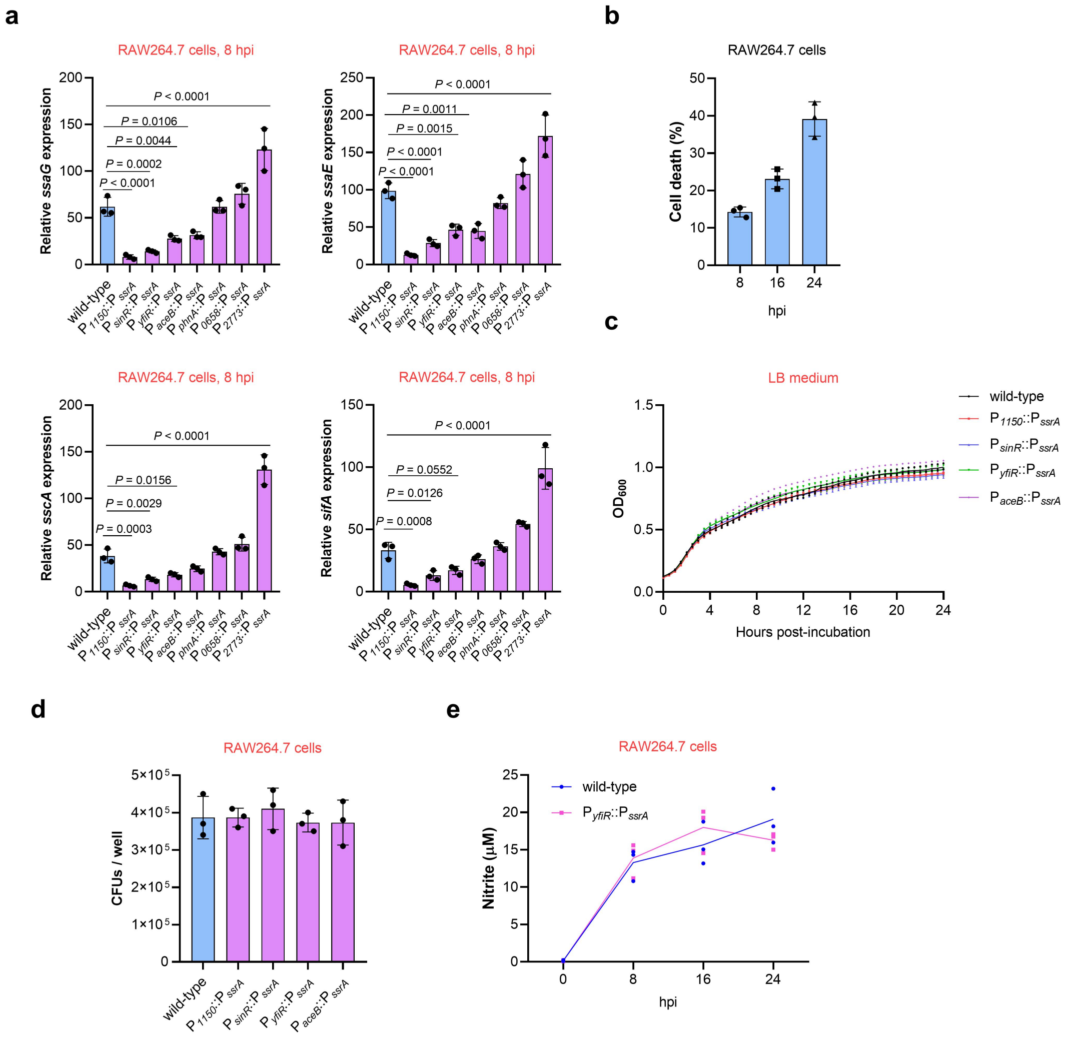

Supplementary Fig. 3 (related to Fig. 3) *S.* Typhimurium SPI-2 expression levels correlate positively with bacterial replication in mouse macrophages and *in vivo* virulence. a, qRT-PCR analysis of *S.* Typhimurium *ssaG, ssaE, sscA* and *sifA* mRNA levels in wild-type and different promoter–replaced strains (n = 3 independent experiments). RNA was extracted from bacteria collected from RAW264.7 cells at 8 hpi. RNA extracted from bacteria in the RPMI-1640 medium was used as a control. **b**, Death of RAW264.7 cells (%) after *S.* Typhimurium infection (n = 3 independent experiments). RAW264.7 cells were infected with wild-type at an MOI of 10, and release of lactate dehydrogenase (LDH) was measured in supernatants at 8, 16, and 24 hpi. **c**, Growth curves of *S.* Typhimurium wild-type and different promoter–replaced

strains in LB medium (n = 3 independent experiments). **d**, Uptake of *S.* Typhimurium wild-type and different promoter–replaced strains by RAW264.7 cells (n = 3 independent experiments). RAW264.7 cells and PMs were infected with different *S.* Typhimurium strains at an MOI of 10. At 15 min post-infection, the infected cells were washed three times with PBS, and intracellular bacteria were released for CFU enumeration. **e**, Nitrite production by RAW264.7 cells left untreated (0 h) or infected with *S.* Typhimurium wild-type or promoter–replaced strain P_{yfiR}::P_{ssrA} (n = 3 independent experiments). Cells were infected at a MOI of 10, and nitrite levels in the supernatant were measured using Griess assays at 0, 8, 16 and 24 hpi. All data are presented as mean ± SD. *P* values were determined using one-way ANOVA (**a,d**), or two-way ANOVA (**e**). hpi, hours post-infection. Source data are included in Supplementary Data 1.

Supplementary Fig. 4

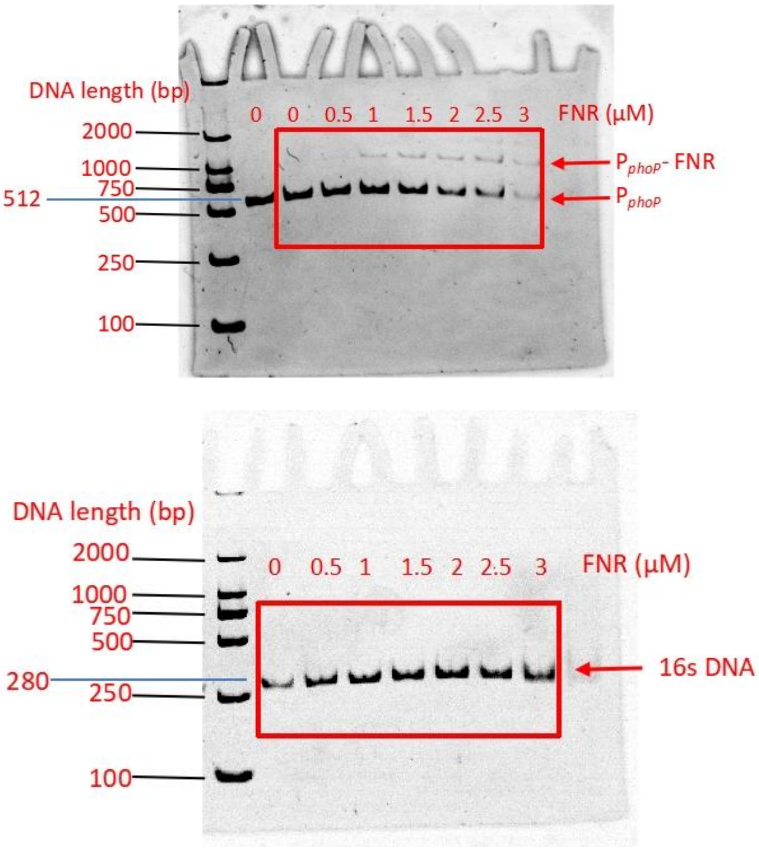

Supplementary Fig. 4 (related to Fig. 5e) Full gels of the EMSA. Boxes highlight lanes used in the Fig. 5e.

Supplementary Fig. 5

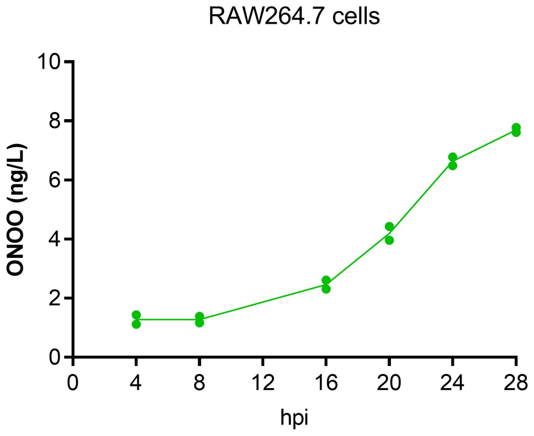

Supplementary Fig. 5 ONOO$^-$ concentrations in RAW264.7 cells infection with *S.* Typhimurium at 4, 8, 16, 20, 24, and 28 hpi. RAW264.7 cells infected with *S.* Typhimurium wild-type at MOI of 10 were lysed at the indicated time points and the supernatant was collected for the measurement of ONOO$^-$ (n = 2 independent experiments). hpi, hours post-infection. Source data are included in Supplementary Data 1.

Supplementary Fig. 6

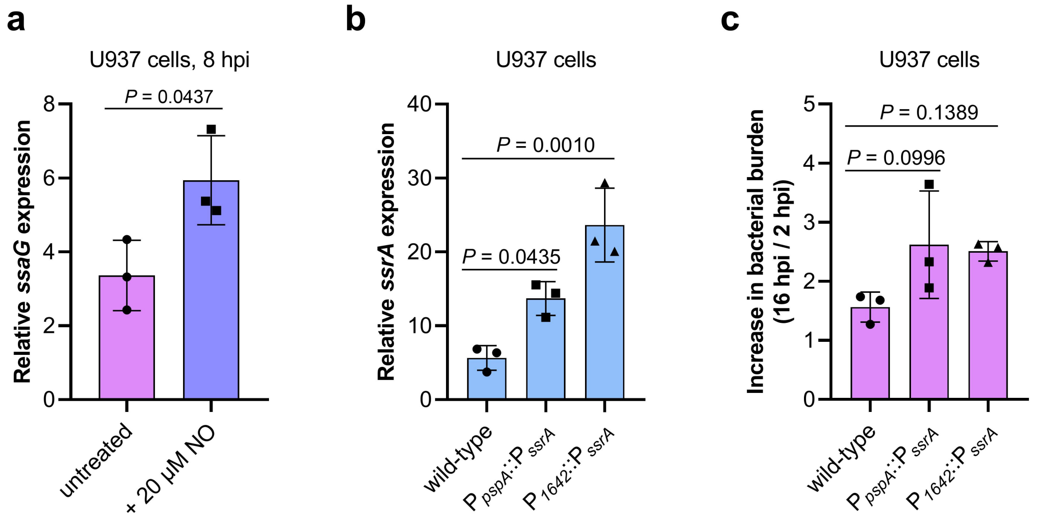

Supplementary Fig. 6 Increasing SPI-2 expression in human U937 cells leads to limited increase in intracellular replication of *S.* Typhimurium. **a**, qRT-PCR analysis of *ssaG* expression in U937 cells at 8 hpi in the presence or absence of 20 µM NO (n = 3 independent experiments). **b,** qRT-PCR analysis of *ssrA* mRNA levels in wild-type and two promoter–replaced strains after infection of U937 cells (n = 3 independent experiments). U937 cells were infected with bacteria at an MOI of 10. RNA was harvested from intracellular bacteria at 8 hpi. Fold changes in *ssrA* gene intracellular expression relative to its expression in RPMI medium are presented. **c,** Increase in bacterial burden of wild-type and two promoter–replaced strains in U937 cells at 16 hpi relative to 2 hpi (n = 3 independent experiments). All data are presented as mean ± SD. *P* values were determined using two-tailed unpaired Student's *t*-test (**a**) or one-way ANOVA (**b,c**). hpi, hours post infection. Source data are included in Supplementary Data 1.

–**Supplementary Tables**

Supplementary Table 1 Expression of SPI-2 genes in different macrophages

Gene	Fold change (RAW264.7–8 h/ control)	Fold change (U937–8 h/ control)	Fold change (RAW264.7–8 h *vs* U937–8 h)	qRT-PCR verification (RAW264.7–8 h *vs* U937–8 h)
ssrB	77.42	13.98	5.54	40.9 vs 13.2
ssrA	64.45	14.46	4.46	26.6 vs 7.4
ssaB	>1240	>195	6.36	
ssaC	360.24	98.78	3.65	
ssaD	191.56	39.79	4.81	
ssaE	>3298	>228	14.46	206.4 vs 20.8
sseA	2782.34	830.96	3.35	
sseB	746.49	107.42	6.95	
sscA	95.33	40.84	2.33	36.3 vs 12.0
sseC	426.72	43.20	9.88	
sseD	87.9394	18.99	4.63	
sseE	227.11	41.48	5.47	
sscB	19.45	8.26	2.36	
sseF	7.82	4.79	1.63	
sseG	2.13	4.18	0.51	
ssaG	671.10	14.62	45.92	
ssaH	80.15	11.90	6.74	
ssaI	723.75	55.71	12.99	
ssaJ	276.06	68.88	4.01	
ssaK	184.49	17.98	10.26	
ssaL	25.49	11.08	2.30	
ssaM	562.24	55.17	10.19	
ssaV	50.12	11.26	4.45	35.3 vs 8.7
ssaN	17.73	6.90	2.57	
ssaO	28.81	2.45	11.77	
ssaP	82.00	3.52	23.30	
ssaQ	20.13	2.66	7.58	
ssaR	98.51	47.53	2.07	
ssaS	26.12	30.04	0.87	
ssaT	264.20	43.31	6.10	
ssaU	94.06	11.62	8.10	
sifA	86.81	21.19	4.10	110.5 vs 20.1
sifB	784.05	54.18	14.47	
sseJ	175.43	27.98	6.27	

Supplementary Table 2 Gene promoters selected for replacement of P_{ssrA}

Gene	Fold change (RAW264.7–8 h/control)
STM14_1150	3
yfiR	6
aceB	12
sinR	14
phnA	18
STM14_0658	81
STM14_2773	455

Supplementary Table 3 Expression levels of the SPI-2 regulators in mouse and human macrophages

	RAW264.7–8 h/ control	U937–8 h/ control
**repressor**		
hns	3.60	0.43
hha	1.68	0.97
ydgT	44.54	1.51
pmrB	1.19	1.49
pmrA	0.30	0.60
**Activator**		
fis	4.76	2.70
IHFa	2.88	1.02
IHFb	0.43	1.02
slyA	4.68	2.59
phoQ	4.25	6.62
phoP	14.22	6.85
envZ	0.53	0.69
ompR	0.32	0.36
hilD	4.35	3.69

Supplementary Table 4 Expression levels of the NO sensors in mouse and human macrophages

Gene	RAW264.7–8 h/ control	U937–8 h/ control
fnr	14.61	3.71
ybaG	1.21	1.23
soxR	3.71	7.51
oxyR	0.31	0.83
nrdR	1.53	2.83
ygaA	0.10	2.29
fur	1.30	0.69
yjeB	4.80	2.64

Supplementary Table 5 Bacterial strains and plasmids used in this study

Plasmid or strain	Genotype or description	Source
**Plasmids**		
pKD46	Red recombinase system under an arabinose–inducible promoter; Ap^R	Lab collection
pKD3	Template plasmid containing the Cm cassette for λ Red recombination; Cm^R	Lab collection
pET28a	T_7 expression vector; Km^R	Lab collection
pWSK129	low–copy–number expression vector; Km^R	Lab collection
pWSK-3×FLAG	pWSK129 carrying 3×FLAG sequence and Cm cassette; Cm^R, Km^R	Lab construction
pFnr	pWSK129 carrying the 14028 *fnr* gene; Km^R	This study
pET-Fnr	pET28a carrying the 14028 *fnr* gene; Km^R	This study
**Strains**		
wild-type	Wild-type S. Typhimurium strain ATCC 14028	ATCC
P_{1150}::P_{ssrA}	14028 strain P_{ssrA} replaced by P_{1150}; Cm^R	This study
P_{sinR}::P_{ssrA}	14028 strain P_{ssrA} replaced by P_{sinR}; Cm^R	This study
P_{yfiR}::P_{ssrA}	14028 strain P_{ssrA} replaced by P_{yfiR}; Cm^R	This study
P_{aceB}::P_{ssrA}	14028 strain P_{ssrA} replaced by P_{aceB}; Cm^R	This study
P_{phnA}::P_{ssrA}	14028 strain P_{ssrA} replaced by P_{phnA}; Cm^R	This study
P_{0658}::P_{ssrA}	14028 strain P_{ssrA} replaced by P_{0658}; Cm^R	This study
P_{2773}::P_{ssrA}	14028 strain P_{ssrA} replaced by P_{2773}; Cm^R	This study
P_{pspA}::P_{ssrA}	14028 strain P_{ssrA} replaced by P_{pspA}; Cm^R	This study
P_{1642}::P_{ssrA}	14028 strain P_{ssrA} replaced by P_{1642}; Cm^R	This study
Δ*fnr*	14028 strain *fnr* gene deleted	This study
Δ*fnr* +pFnr	Δ*fnr* containing plasmid pFnr; Km^R	This study
WT *fnr*-FLAG	14028 strain *fnr* gene tagged with 3×FLAG tag; Cm^R	This study
