# Supplementary material for: Nitric oxide is a host cue for Salmonella Typhimurium systemic infection in mice
Source: Commun Biol. 2023 May 9;6:501. doi: 10.1038/s42003-023-04876-1 (PMC10169850; doi:10.1038/s42003-023-04876-1)
Supplement: Supplementary file 3 — Description of Additional Supplementary Data [file 42003_2023_4876_MOESM3_ESM.pdf]

Description of Additional Supplementary Files

File name: Supplementary Data 1

Description: Primers used in this study

File name: Supplementary Data 2

Description: The source data behind the graphs in the paper
