# Supplementary material for: Nitric oxide is a host cue for Salmonella Typhimurium systemic infection in mice
Source: Commun Biol. 2023 May 9;6:501. doi: 10.1038/s42003-023-04876-1 (PMC10169850; doi:10.1038/s42003-023-04876-1)
Supplement: Supplementary file 6 — Reporting summary [file 42003_2023_4876_MOESM6_ESM.pdf]

nature portfolio

Corresponding author(s): Lu Feng

Last updated by author(s): Apr 19, 2023

Reporting Summary

Nature Portfolio wishes to improve the reproducibility of the work that we publish. This form provides structure for consistency and transparency in reporting. For further information on Nature Portfolio policies, see our Editorial Policies and the Editorial Policy Checklist.

Statistics

For all statistical analyses, confirm that the following items are present in the figure legend, table legend, main text, or Methods section.

n/a	Confirmed	
☐	☒	The exact sample size (*n*) for each experimental group/condition, given as a discrete number and unit of measurement
☐	☒	A statement on whether measurements were taken from distinct samples or whether the same sample was measured repeatedly
☐	☒	The statistical test(s) used AND whether they are one- or two-sided<br>*Only common tests should be described solely by name; describe more complex techniques in the Methods section.*
☒	☐	A description of all covariates tested
☐	☒	A description of any assumptions or corrections, such as tests of normality and adjustment for multiple comparisons
☐	☒	A full description of the statistical parameters including central tendency (e.g. means) or other basic estimates (e.g. regression coefficient) AND variation (e.g. standard deviation) or associated estimates of uncertainty (e.g. confidence intervals)
☐	☒	For null hypothesis testing, the test statistic (e.g. *F*, *t*, *r*) with confidence intervals, effect sizes, degrees of freedom and *P* value noted<br>*Give P values as exact values whenever suitable.*
☒	☐	For Bayesian analysis, information on the choice of priors and Markov chain Monte Carlo settings
☒	☐	For hierarchical and complex designs, identification of the appropriate level for tests and full reporting of outcomes
☒	☐	Estimates of effect sizes (e.g. Cohen's *d*, Pearson's *r*), indicating how they were calculated

Our web collection on statistics for biologists contains articles on many of the points above.

Software and code

Policy information about availability of computer code

Data collection	Sequencing data was collected by Illumina HiSeq 2000 platform.
Data analysis	GraphPad Prism 8.0.1; ZEN 2.3 (blue edition); Bowtie (version 2.2.3); HTSeq (version 0.6.1); DESeq2.

For manuscripts utilizing custom algorithms or software that are central to the research but not yet described in published literature, software must be made available to editors and reviewers. We strongly encourage code deposition in a community repository (e.g. GitHub). See the Nature Portfolio guidelines for submitting code & software for further information.

Data

Policy information about availability of data

All manuscripts must include a data availability statement. This statement should provide the following information, where applicable:

- Accession codes, unique identifiers, or web links for publicly available datasets
- A description of any restrictions on data availability
- For clinical datasets or third party data, please ensure that the statement adheres to our policy

The RNA-seq data acquired in this study are available in the NCBI Sequence Read Archive (SRA, PRJNA915482, https://www.ncbi.nlm.nih.gov/sra/PRJNA915482). Source data are provided in Supplementary Data 1.

Human research participants

Policy information about studies involving human research participants and Sex and Gender in Research.

Reporting on sex and gender	*Use the terms sex (biological attribute) and gender (shaped by social and cultural circumstances) carefully in order to avoid confusing both terms. Indicate if findings apply to only one sex or gender; describe whether sex and gender were considered in study design whether sex and/or gender was determined based on self-reporting or assigned and methods used. Provide in the source data disaggregated sex and gender data where this information has been collected, and consent has been obtained for sharing of individual-level data; provide overall numbers in this Reporting Summary. Please state if this information has not been collected. Report sex- and gender-based analyses where performed, justify reasons for lack of sex- and gender-based analysis.*
Population characteristics	*Describe the covariate-relevant population characteristics of the human research participants (e.g. age, genotypic information, past and current diagnosis and treatment categories). If you filled out the behavioural & social sciences study design questions and have nothing to add here, write "See above."*
Recruitment	*Describe how participants were recruited. Outline any potential self-selection bias or other biases that may be present and how these are likely to impact results.*
Ethics oversight	*Identify the organization(s) that approved the study protocol.*

Note that full information on the approval of the study protocol must also be provided in the manuscript.

Field-specific reporting

Please select the one below that is the best fit for your research. If you are not sure, read the appropriate sections before making your selection.

☒ Life sciences ☐ Behavioural & social sciences ☐ Ecological, evolutionary & environmental sciences

For a reference copy of the document with all sections, see nature.com/documents/nr-reporting-summary-flat.pdf

Life sciences study design

All studies must disclose on these points even when the disclosure is negative.

Sample size	The in vitro experiments were performed in duplicate and repeated at least three times ($n \geq 3$), unless specifically stated. Mouse virulence assays were conducted twice with at least 2 mouse ($n \geq 2$) in each injection group, and the combined data for the two experiments was used for statistical analysis.
Data exclusions	No data were excluded from the analyses.
Replication	All the reported experiments were reproducible. Data reproducibility was confirmed by two or three independent experiments.
Randomization	Mice were randomized into the different groups with same gender and similar weight.
Blinding	Sequencing of cDNA libraries was done blindly by NovogeneCo.,Ltd. (Beijing, China), which also provided the statistical analysis. Other experiments were not done blindly. Most experiments were conducted by at least two different researchers who have not known the situation and results of the study in advance and repeated on at least two independent days.

Reporting for specific materials, systems and methods

We require information from authors about some types of materials, experimental systems and methods used in many studies. Here, indicate whether each material, system or method listed is relevant to your study. If you are not sure if a list item applies to your research, read the appropriate section before selecting a response.

Materials & experimental systems

n/a	Involved in the study
	☒ Antibodies
	☒ Eukaryotic cell lines
☒	Palaeontology and archaeology
	☒ Animals and other organisms
☒	Clinical data
☒	Dual use research of concern

Methods

n/a	Involved in the study
☒	ChIP-seq
☒	Flow cytometry
☒	MRI-based neuroimaging

Antibodies

Antibodies used	Anti-Salmonella typhimurium LPS antibody cat.ab8274. Abcam. 1:100 dilution used for immunofluorescence.<br>Goat anti-mouse IgG (FITC) cat.ab6785. Abcam. 1:200 dilution used for immunofluorescence.<br>Anti-FLAG mouse monoclonal antibody cat.F1804. Sigma. 1:1,000 dilution used for chromatin immunoprecipitation.
Validation	All antibodies were obtained commercially and were tested and validated by the respective company. All antibodies had validation statement provided on the website of the manufacturer.<br>Anti-Salmonella typhimurium LPS antibody: mouse monoclonal to Salmonella typhimurium LPS; suitable for WB, Dot blot, ELISA, ICC/IF, and IHC-Fr; reacts with Salmonella typhimurium.<br>Anti-FLAG mouse monoclonal antibody: mouse monoclonal to FLAG-tag; suitable for WB, IP, ICC/IF, and IHC-Fr; reacts with FLAG-tag (DYKDDDDK).

Eukaryotic cell lines

Policy information about cell lines and Sex and Gender in Research

Cell line source(s)	The mouse macrophage cell line RAW264.7 (ATCC TIB71), human macrophage cell line U937 (ATCC CRL-1593.2) and THP-1 (ATCC TIB-22) were purchased from the Shanghai Institute of Biochemistry and Cell Biology of the Chinese Academy of Sciences (Shanghai, China).
Authentication	All the cell lines of the Shanghai Institute of Biochemistry and Cell Biology are originated from ATCC. ATCC authenticates its cell lines through morphology, karyotyping, and STR analyses, thus RAW264.7, U937, and THP-1 cells were not authenticated after receipt. Regular inspection of cell culture for coherent morphology with ATCC source images was routinely performed.
Mycoplasma contamination	Cells routinely tested negative for mycoplasma contamination.
Commonly misidentified lines<br>(See ICLAC register)	No commonly misidentified cell lines were used.

Animals and other research organisms

Policy information about studies involving animals; ARRIVE guidelines recommended for reporting animal research, and Sex and Gender in Research

Laboratory animals	Wild-type C57BL/6 mice were purchased from Beijing Vital River Laboratory Animal Technology Co. Ltd (Beijing, China). Congenic iNOS-/- mice with a C57BL/6 background were purchased from the Jackson Laboratory (USA). All mice were raised in a specific sterile environment in compliance with the scientific guidelines for animal research.
Wild animals	No wild animals were used in the study.
Reporting on sex	Sex was not considered in study design.
Field-collected samples	No field-collected samples were employed in this study.
Ethics oversight	All animal experiments were approved by the Institutional Animal Care and Use Committee (IACUC) at Nankai University, Tianjin, China (IACUC number: 2018050601).

Note that full information on the approval of the study protocol must also be provided in the manuscript.
